# The neural network basis of altered decision‐making in patients with amyotrophic lateral sclerosis

**DOI:** 10.1002/acn3.51185

**Published:** 2020-10-22

**Authors:** Kazunori Imai, Michihito Masuda, Hirohisa Watanabe, Aya Ogura, Reiko Ohdake, Yasuhiro Tanaka, Toshiyasu Kato, Kazuya Kawabata, Yuichi Riku, Kazuhiro Hara, Ryoichi Nakamura, Naoki Atsuta, Epifanio Bagarinao, Kentaro Katahira, Hideki Ohira, Masahisa Katsuno, Gen Sobue

**Affiliations:** ^1^ Department of Neurology Nagoya University Graduate School of Medicine Nagoya Japan; ^2^ Department of Neurology Fujita Health University Toyoake Japan; ^3^ Brain and Mind Research Center Nagoya University Nagoya Japan; ^4^ Department of Psychology Nagoya University Graduate School of Informatics Nagoya Japan

## Abstract

**Objective:**

Amyotrophic lateral sclerosis (ALS) is a multisystem disorder associated with motor impairment and behavioral/cognitive involvement. We examined decision‐making features and changes in the neural hub network in patients with ALS using a probabilistic reversal learning task and resting‐state network analysis, respectively.

**Methods:**

Ninety ALS patients and 127 cognitively normal participants performed this task. Data from 62 ALS patients and 63 control participants were fitted to a Q‐learning model.

**Results:**

ALS patients had anomalous decision‐making features with little shift in choice until they thought the value of the two alternatives had become equal. The quantified parameters (Pαβ) calculated by logistic regression analysis with learning rate and inverse temperature well represented the unique choice pattern of ALS patients. Resting‐state network analysis demonstrated a strong correlation between Pαβ and decreased degree centrality in the anterior cingulate gyrus and frontal pole.

**Interpretation:**

Altered decision‐making in ALS patients may be related to the decreased hub function of medial prefrontal areas.

## Introduction

Amyotrophic lateral sclerosis (ALS) is an adult‐onset neurodegenerative disorder characterized by upper and lower motor neuron involvement.^1^ Although extra‐motor cognitive and behavioral impairment were considered atypical clinical features in early descriptions of ALS, recent studies demonstrated that approximately 50% of patients with ALS suffer from frontotemporal dysfunction.^2^ Furthermore, almost all patients with sporadic ALS and more than half of those with frontotemporal dementia (FTD) had TAR DNA‐binding protein 43 (TDP‐43)‐positive ubiquitinated cytoplasmic inclusions.^1^, ^2^, ^3^, ^4^

While ALS and FTD are certainly on a spectrum, most ALS patients do not meet the FTD criteria and they are not consecutive in time. However, previous studies demonstrated that ALS patients who do not meet the FTD criteria show abnormalities not only in the frontotemporal cortex but also in the basal ganglia, including the head of the caudate nucleus and its networks.^5^, ^6^, ^7^ Although frontostriatal circuits are associated with decision‐making processes^8^ and patients with FTD show various errors in decision‐making,^9^, ^10^, ^11^ there is limited available evidence regarding the features of decision‐making, particularly its network basis, in patients with ALS.

The probabilistic reversal learning (PRL) task is widely used to assess deficits in decision‐making associated with the caudate nucleus, orbitofrontal cortex, and medial cingulate cortex.^8^ The PRL task is composed of two stages, that is, acquisition learning and reversal learning. Due to its stochastic nature, performance is optimally achieved by incremental learning of the action–outcome contingency over many trials. Reversal learning needs a flexible change in a previously established stimulus–response association when the prior response is no longer rewarding.

PRL also includes representative models of reinforcement learning (RL), which is a behavioral process to learn the values of actions to improve future outcomes using trial and error.^12^, ^13^ RL has two critical parameters: learning rate (alpha), which represents the extent to which old values are updated by newly acquired information, and inverse temperature (beta), known as the degree to which value estimates influence choice; high inverse temperatures indicate that individuals tend to select the higher‐value option, while low inverse temperatures indicate that value differences between options govern choices to a lesser extent.

In the present study, we investigated alterations in PRL findings in patients with ALS and their underlying network basis using resting‐state functional MRI (RS‐fMRI). We used intrinsic connectivity contrast (ICC),^14^ which is a whole‐brain voxel‐based hypothesis‐free analysis based on graph theory that provides a correlation map without prior information or assumptions.^15^

## Methods

### Participants

Patients with ALS were recruited at the Department of Neurology at Nagoya University (March 2015 to December 2018). All patients fulfilled the El Escorial revised criteria for probable laboratory‐supported or probable ALS.^16^ A board‐certificated neurologist (W.H.) and trained speech therapist (O.R.) conducted semi‐structured clinical interviews concerning the FTD criteria.^17^, ^18^ Two patients with ALS met the criteria for behavioral variant FTD.

We also evaluated cognitively normal participants who did not show any cognitive impairment (Addenbrooke's Cognitive Examination Revised [ACE‐R] ≥ 89) or neurological and psychological diseases. Based on the Fazekas hyperintensity rating system, we could not find white matter abnormalities characterized by hyperintensities more severe than grade 2 in T2‐weighted MR images.^19^

All participants in the present study had adequate vision (with or without eyeglasses) to perform the PRL task.

This study was conducted according to the Ethical Guidelines for Medical and Health Research Involving Human Subjects endorsed by the Japanese government and approved by the Ethical Review Committee of Nagoya University Graduate School of Medicine. We obtained written informed consent from patients with ALS and cognitively normal participants.

### Cognitive assessments

To assess general cognitive function, the Mini‐Mental State Examination (MMSE), ACE‐R,^20^, ^21^ Frontal Assessment Battery (FAB), Stroop test, digit span (forward and backward), and word fluency (letter and semantic) were performed to assess detailed executive function in patients with ALS.

### PRL task

The PRL task comprised 120 trials. In each trial, the participants were presented with two abstract line drawings on the left and right sides of the screen. The presentation side was randomized for each trial. The participants were asked to select one drawing by pressing a key within 1000 ms, and were consequently presented with a reward or loss. If the participant did not select a stimulus within the presentation time window, the message “Time‐up” appeared and the next trial was initiated. During the first 60 trials, one drawing had an advantageous option; the reward/loss frequency ratio was 80:20. The other drawing had a disadvantageous option; the reward/loss frequency ratio was 20:80. After 60 trials, the contingencies were reversed without any instruction to the participants (Supplementary Data 1). Before starting the examination, we set up a practice task to see if the participants understood the task. After the examination, we asked the participants how they had made their choice and reconfirmed that they had understood the task. The paradigm used here was based on previous studies.^22^

We excluded participants whose score during the acquisition phase was over 1.5 standard deviations below the mean score of cognitively normal participants because they might not have understood the task. We also excluded cognitively normal participants whose score of the reversal phase was over 1.5 standard deviations below the mean score to only select participants who performed the task successfully.

### Learning models

We used Q‐learning, win–stay, lose–shift (WSLS), and random choice models to classify choice behavior.^8^ When analyzing decision‐making behavior through RL, the elimination of subjects who randomly select their choices is generally used.^23^, ^24^ In decision‐making tasks, including the PRL task, it is well known that some participants make decisions based on the WSLS model instead of the RL model. The WSLS model is only sensitive to the outcome of the previous choice, and the mathematical formulas used for analysis are different.^13^, ^25^, ^26^ It is also necessary to exclude cases who adopt the WSLS model when analyzing decision‐making behavior.

The Q‐learning model is one of the most famous computational RL models. This model updates action values based on the Rescorla–Wagner model.^13^, ^25^ After the participant chooses a stimulus and feedback is presented, the estimated action values are updated as follows:

$$Q_{s}\left(t+1\right)=Q_{s}\left(t\right)+\alpha \left(R\left(t\right)‐Q_{s}\left(t\right)\right)$$

where $Q_{s}\left(t\right)$ is the estimated action value for stimulus *s* on trial *t*, and *R(t)* is the reward value of the choice on trial *t*. Given the estimated values are set, the probability of choosing stimulus 1 in the next trail is determined as the soft‐max function below:

$$P_{\left(a(t)=1\right)}=\frac{1}{1+e^{‐\beta (Q_{1}\left(t\right)‐Q_{2}\left(t\right))}}$$

where *a(t)* represents the participant’s choice on trial *t*.

The probability of choosing stimulus 2 is calculated by the following equation:

$$P_{\left(a(t)=2\right)}\left(t\right)=1‐P_{\left(a\left(t\right)=1\right)}\left(t\right).$$

We adopted the maximum‐likelihood approach to fit the parameter set of alpha and beta to the participant’s choice behavior. The log‐likelihood for the entire trial is as follows:

$$\text{LL}=\sum_{t=1}^{T}logP_{a(t)}\left(t\right).$$

The optima function in R was employed to find the parameter set that produced the highest log‐likelihood.

The WSLS model in the present study had two parameters. The first parameter represented the probability of staying with the same option on the next trial if a reward was provided:

$$P_{\left(stay|win\right)}=P_{\left(a(t+1)=a(t)|R(t)=1\right)}$$

The probability of switching to another option following a win trial was $1‐P_{\left(stay|win\right)}$. The second parameter represented the probability of shifting to another option on the next trial if a reward was not provided:

$$P_{\left(lose|shift\right)}=P_{\left(a(t+1)\neq a(t)|R(t)=0\right)}$$

The probability of staying with an option following a loss trial was $1‐P_{\left(shift|loss\right)}$.^26^

In the random choice model, each choice of options was made randomly and in equal frequency.

To clarify which strategy the participant adopted, we compared the goodness‐of‐fit of the three models with the best‐fit parameter set. We computed Akaike’s information criterion (AIC), as shown by the following equation^27^:

AIC=‐2L+2k

where *k* is the number of free parameters (two for the standard Q‐learning model, two for the WSLS model, and zero for the random choice model). We regarded the model with the smallest AIC value as the strategy the participant adopted, and targeted participants who employed the Q‐learning model.

### Logistic regression analysis

Logistic regression analysis of alpha and beta values showed the extent to which the participant’s choice behavior was characteristic to ALS compared with the control group. This estimated logistic regression equation was designated as “P_αβ_” in the current study. By using receiver operating characteristic (ROC) analysis, we calculated the value of P_αβ_ that most efficiently discriminated between the ALS and control groups. Based on the value of P_αβ_, patients with ALS were classified into two groups: patients with anomalous choice behavior and patients with normal choice behavior. We compared ALS patients who had anomalous choice behavior with those who had normal choice behavior, and assessed the clinical features in ALS patients with anomalous choice behavior.

### Analysis of RS‐fMRI

RS‐fMRI was used to identify the underlying neural networks linked to specific choice behavior in patients with ALS. All MRI scans were performed using a Siemens Magnetom Verio (Siemens, Erlangen, Germany) 3.0‐T scanner with a 32‐channel head coil at the Brain and Mind Research Center. High‐resolution T1‐weighted images (T1‐WI) were acquired using the following parameters: repetition time (TR) = 2.5 s, echo time (TE) = 2.48 ms, 192 sagittal slices with 1‐mm thickness, field of view (FOV) = 256 mm, 256 × 256 matrix size, and an in‐plane voxel resolution of 1 × 1 mm^2^. Eight‐minute closed eyes resting‐state functional images were obtained with echo‐planar imaging using the following parameters: TR = 2.5 s, TE = 30 ms, 39 transversal slices with a 0.5‐mm interslice interval and 3‐mm thickness, FOV = 192 mm, 64 × 64 matrix dimension, flip angle = 80°, and 198 volumes.

Functional MRI data were preprocessed using the default pipeline as the standard procedure implemented in Conn Functional Connectivity Toolbox version 18b,^28^ using MATLAB. The first five volumes of each participant’s data were discarded to remove initial image inhomogeneity. Then, each participant’s images were realigned, unwarped to remove dynamic EPI distortions,^29^ slice‐time corrected, co‐registered to the bias corrected T1‐WI, segmented and normalized to Montreal Neurological Institute coordinates, resampled to an isotropic voxel resolution of 2 × 2 × 2 mm^3^, and smoothed using an 8‐mm FWHM Gaussian filter. Moreover, the principal component‐based noise‐correction CompCor approach was applied to remove the BOLD signal noise associated with white matter and cerebrospinal fluid. Band‐pass filtering was performed with a frequency window of 0.008–0.09 Hz. Artifact detection tools (ART)‐based outlier detection and scrubbing were performed to eliminate the effects of head motion.

### ICC analysis

In graph theory, “degree centrality” represents the number of direct connections between a given region and the rest of the brain. By measuring degree centrality, ICC analysis detects hub regions.^14^ We calculated the ICC‐power (ICC‐p) value that could be interpreted as a “weighted” degree centrality, a network measure representing the number of connections between a given voxel and the rest of the brain, obtained with the connectivity threshold set to 0. We performed correlation analysis between the ICC‐p value and P_αβ_ using the CONN toolbox considering age, sex, and education history as nuisance covariates. Statistical significance was set at a height threshold of *P* < 0·005, FDR‐corrected cluster‐size threshold of *P* < 0·05, and one‐sided negative contrast to reveal network dysfunction. We calculated the mean ICC‐p value of the cluster with significantly different ICC‐*P* values.

We subsequently performed seed‐to‐voxel analysis to identify the origin of the alterations in degree centrality. First, all regions with significantly different ICC‐p values were extracted as the regions of interest for seeds. The average filtered BOLD signal in each seed was evaluated, and its bivariate correlation with the BOLD signal in all other voxels in the brain was computed. Then, we conducted correlation analysis on the calculated connectivity and P_αβ_. The results were assessed with a cluster‐forming height threshold of *P* < 0.005 and FDR‐corrected cluster‐size threshold of *P* < 0.05.

### Statistical analyses

Clinical backgrounds and cognitive examinations were compared using the Mann–Whitney or chi‐squared test. The threshold of statistical significance was set at *P* < 0.05. Statistical analyses were performed using the Statistical Package for the Social Sciences (SPSS) version 24 (SPSS, Inc., Chicago, IL, USA).

## Results

### Specific choice behavior in patients with ALS

**Figure 1:**
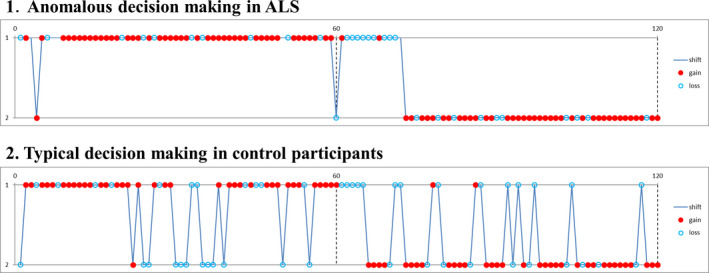
Examples of anomalous choice behavior in cognitively normal participants and patients with amyotrophic lateral sclerosis (ALS). Some ALS patients followed a rational method of choice behavior, in which after a short or no trial and error, they learned to choose the advantageous stimulus with minimum selection of the disadvantageous stimulus. When the contingencies changed, they shifted to a new advantageous stimulus with minimum selection of the disadvantageous stimulus. Conversely, typical decision‐making in control participants had some exploration choices. The red dot indicates when the participants received a reward feedback signal (“Atari” in Japanese). The blue dot indicates when the participants did not receive a reward. The blue line indicates a change in participant choice. The absence of a sign indicates that the participants did not select a stimulus within the presentation time window. Akaike’s information criterion showed anomalous decision‐making in patients with ALS had the lowest value in the Q‐learning model (Q‐learning = 33, WSLS = 46, random choice = 158).

We performed the PRL task in 127 cognitively normal people and 90 patients with ALS. Clinical backgrounds and cognitive examinations are shown in Supplementary Data 2. Although there were no significant differences in the scores of the PRL tasks and ratio of strategies classified by AIC, we found some ALS patients demonstrated a unique choice behavior (Figure 1), that is, they rarely changed their choice, with few switches in the early stage of the acquisition and reversal phases. Few cognitively normal participants showed such a pattern. Thus, we selected cognitively normal participants with typical choice behavior as control subjects and compared them with ALS patients, to reveal the choice behavior features of ALS patients with unique choice behavior.

First, we excluded 19 cognitively normal participants who scored below 1.5 standard deviations from the mean score during the acquisition and/or reversal phase (score at acquisition phase < 28, score at reversal phase < 20) since they could not perform the PRL task appropriately. Second, we classified their strategies of choice behavior into Q‐learning, WSLS, and random choice models, using AIC. Since the unique choice behavior of patients with ALS had the lowest AIC value of Q‐learning among the three models, we selected 62 from 90 patients with ALS and 66 of 108 control participants who adopted RL (Q‐learning model) and evaluated the parameters in the Q‐learning model. Finally, we excluded three control participants who showed abnormal inverse temperature with scores over 3 standard deviations from the mean score (Supplementary Data 3).

### Demographic and cognitive features of participants with the Q‐learning model

Choice data from 62 patients with ALS and 63 control participants were best fitted using the Q‐learning model. Clinical backgrounds and results of the PRL task are shown in Table 1. Although there was no significant difference in age, the ALS group exhibited significantly lower education and MMSE and ACE‐R scores. In the PRL task, there were no significant differences in total score, score at acquisition phase, or score at reversal phase between the ALS and control participants. Although the alpha value tended to be lower in the ALS group, it was not significantly different from the control group. The mean PRL beta value was significantly higher in patients with ALS compared to controls.

**Table 1:** Clinical features, cognitive examinations, and probabilistic reversal learning (PRL) task results of patients with amyotrophic lateral sclerosis (ALS) and control participants.

	Control	ALS	*P*‐value
N	63	62	‐
Age (y)	63.4 (9.8)	66.0 (8.9)	N.S. (0.152)
Sex (men: women)	24:39	40:21	0.002
Education (years)	14.1 (2.2)	12.1 (2.6)	<0.001
Clinical phenotype (spinal: bulbar: others)	‐	44:16:2	‐
Disease duration (years)	‐	1.9 (1.4)	‐
ALSFRS‐R	‐	40.9 (4.1)	‐
MMSE	29.2 (1.1)	27.7 (2.1)	<0.001
ACE‐R Total score	97.3 (2.6)	89.0 (8.8)	<0.001
ACE‐R Orientation/attention	17.9 (0.3)	17.4 (1.3)	0.002
ACE‐R Memory	24.7 (1.6)	20.4 (4.3)	<0.001
ACE‐R Fluency	13.7 (0.6)	12.2(2.4)	<0.001
ACE‐R Language	25.2 (1.0)	23.8 (2.1)	<0.001
ACE‐R Visuospatial abilities	15.7 (0.7)	15.2 (1.0)	0.004
Total score in PRL task	74.3 (7.6)	72.4 (9.4)	N.S. (0.229)
Score at acquisition phase	41.7 (4.2)	40.9 (6.4)	N.S. (0.980)
Score at reversal phase	32.6 (5.8)	31.5 (8.2)	N.S. (0.692)
Alpha	0.4 (0.3)	0.3 (0.3)	N.S. (0.197)
Beta	4.0 (1.7)	7.0 (7.1)	0.007

Data are shown as mean ± standard deviation (SD). Age, years of education, and scores in cognitive examinations and the PRL task were compared by Mann–Whitney analysis. Sex was compared by the chi‐squared test. The statistical significance threshold was set at *P* < 0.05. ACE‐R, Addenbrooke’s Cognitive Examination revised; ALSFRS‐R, ALS Functional Rating Scale Revised; MMSE, Mini‐Mental State Examination; N.S., not significant.

### Features of choice behavior in patients with ALS

**Figure 2:**
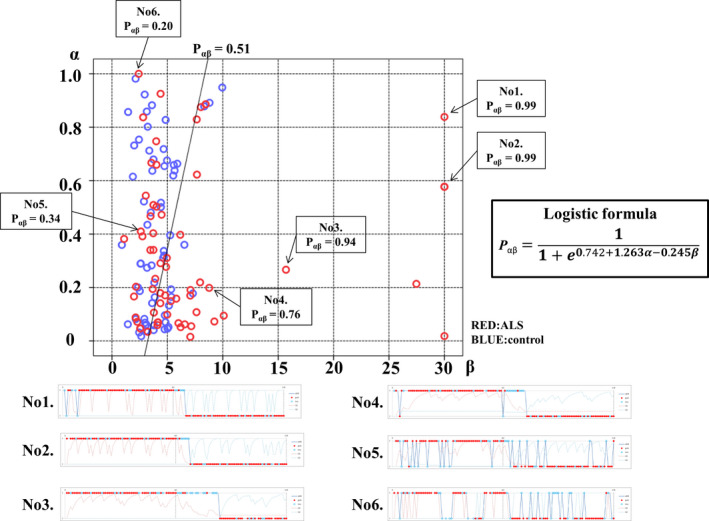
Logistic regression analysis using alpha and beta values. Logistic regression analysis using Q‐learning parameters (alpha and beta) demonstrated that P_αβ_ reflects the specificity of choice behavior in patients with amyotrophic lateral sclerosis (ALS). P_αβ_ was calculated using the following equation: $P\alpha \beta =\frac{1}{1+e^{0.742+1.263\alpha ‐0.245\beta }}$. A higher P_αβ_ was associated with more ALS‐specific choice behavior. Examples of choice behavior are shown in 1–6 (1–5 showed anomalous choice behavior; 6 showed typical choice behavior). The line (*P* = 0.512) demonstrates ROC analysis results, which determined the most effective division between the ALS and control groups.

Figure 1 shows a representative specific choice pattern, with low exploration and high exploitation, observed in patients with ALS, with high beta values, but relatively variable alpha values. However, ALS patients who had a mild to moderate increase in beta values (from 5 to 10) showed lower alpha values than controls. Besides, there was no significant correlation between alpha and beta values in patients with ALS. Thus, we applied logistic regression analysis to predict the probability of ALS (P_αβ_) based on the two parameters concerning choice behavior. According to AIC, P_αβ_ yielded stronger explanatory power than those using alpha or beta values alone (AIC alpha = 174.69, AIC beta = 163.78, AIC alpha and beta = 162.12). A higher P_αβ_ well represented the unique choice pattern observed in patients with ALS (Figure 2). A P_αβ_ cutoff value of 0.512 provide optimal separation between ALS patients and controls in ROC analysis (area under the ROC curve, 0.666 [95% confidence interval 0.571–0.761]; sensitivity, 0.565; and specificity, 0.762). There were no significant correlations between P_αβ_ and age, education, disease type, disease duration, disease severity (ALS Functional Rating Scale Revised [ALSFRS‐R]), and other scores of cognitive tests in patients with ALS (Supplementary Data 4).

### Clinical characteristics and ALS‐specific choice behavior

Using a P_αβ_ cutoff value of 0.512, 35 out of 62 ALS patients were classified into the ALS anomalous choice behavior group. In a comparison of ALS patients who had anomalous choice behavior with those who had typical choice behavior, there were no significant differences in clinical backgrounds and conventional cognitive examinations. In the PRL task, ALS patients with anomalous choice behavior exhibited significantly higher scores at the acquisition phase, lower alpha values, and higher beta values (Supplementary Data 5).

In the 35 patients with ALS who had anomalous choice behavior, the total score of the PRL task was significantly correlated with alpha, MMSE, and ACE‐R. Furthermore, the score at the acquisition phase was significantly correlated with MMSE. The score at the reversal phase was significantly correlated with alpha and MMSE (Supplementary Data 6).

### Functional connectivity changes associated with ALS‐specific choice behavior

We performed RS‐fMRI in 34 patients with ALS and 33 age‐ and sex‐matched control participants. There were no significant differences in clinical backgrounds, PRL scores, parameters, and P_αβ_ of ALS patients who underwent MRI and those who did not undergo MRI. ALS patients who underwent MRI had higher ACE‐R scores than those who did not (Supplementary Data 7).

**Figure 3:**
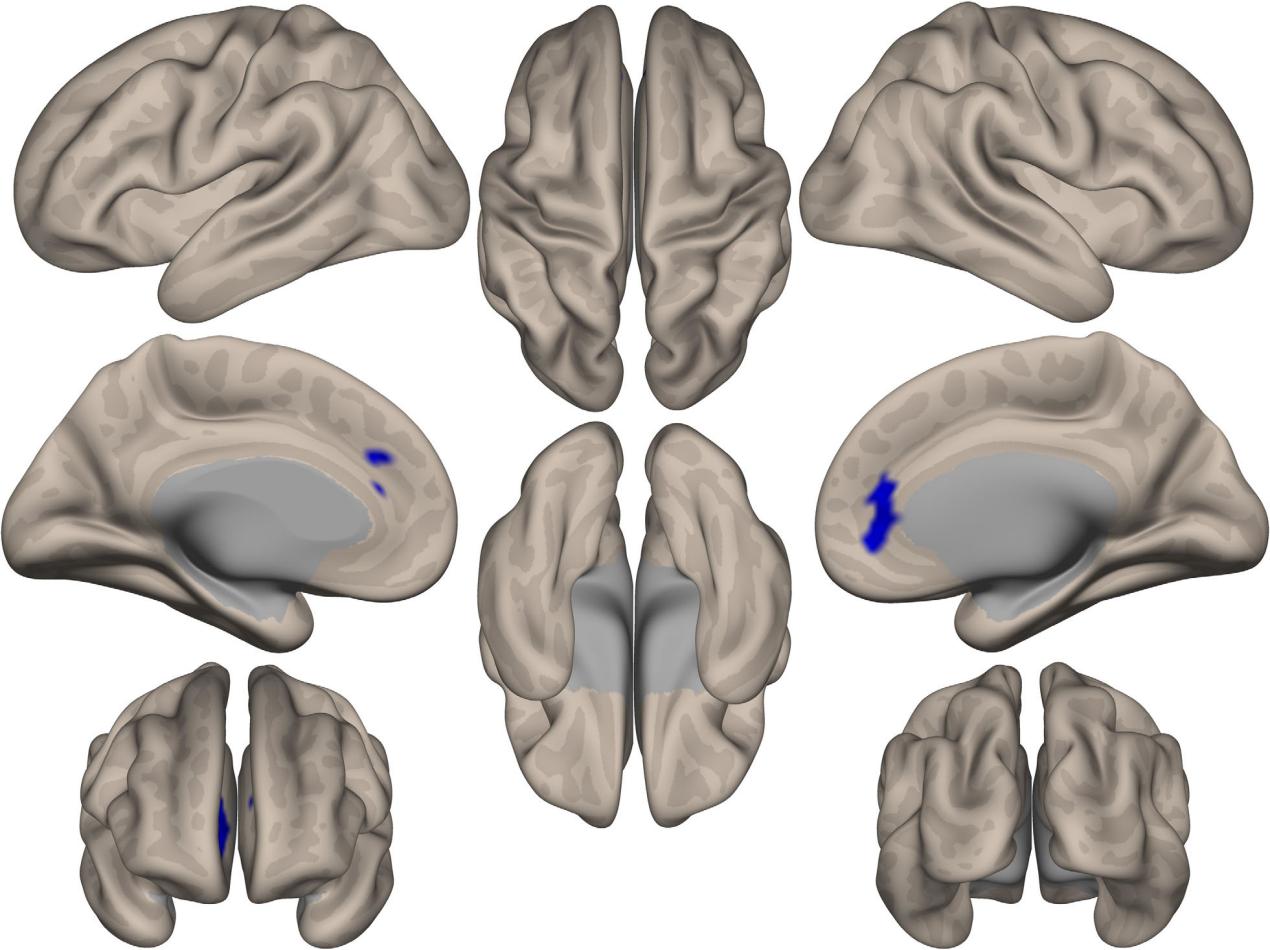
Decreased degree centrality associated with P_αβ_ in patients with amyotrophic lateral sclerosis (ALS). Analysis of resting‐state functional magnetic resonance imaging using intrinsic connectivity contrast demonstrated that the P_αβ_ was associated with decreased degree centrality at the medial prefrontal cortex. This area consisted of the anterior cingulate gyrus and frontal pole in patients with ALS. The results were assessed using a cluster‐forming height threshold of *P* < 0.01 and family‐wise error‐corrected cluster‐size threshold of *P* < 0.05.

**Figure 4:**
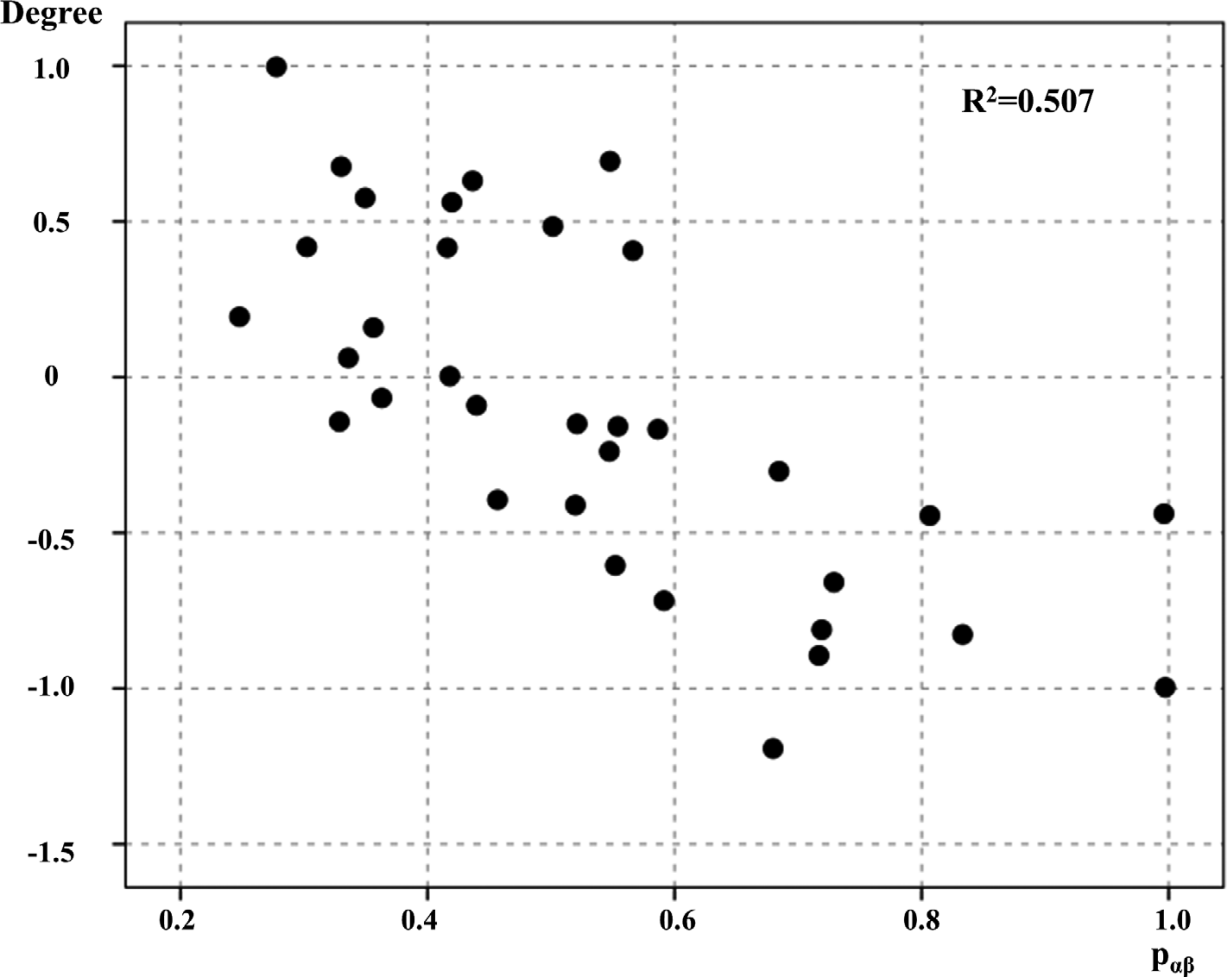
Correlation between degree centrality of regions of interest and P_αβ_. The correlation between the Z score of degree centrality and P_αβ_ are shown. The Z score of degree centrality correlates with P_αβ_ in patients with amyotrophic lateral sclerosis (*r* = −0.712, *P* < 0.001).

ICC analysis demonstrated that P_αβ_ was associated with decreased degree centrality in the region of the anterior cingulate gyrus and frontal pole (Figure 3). Only the ALS group showed a significant correlation between the degree centrality Z‐score in this region and P_αβ_ score (*r* = −0.712, p < 0.001; Figure 4). Seed‐based analysis from the region of the anterior cingulate gyrus and frontal pole revealed that patients with ALS had decreased functional connectivity with the paracingulate gyrus, frontal medial cortex, anterior cingulate gyrus, frontal pole, subcallosal cortex, and superior frontal gyrus (Supplementary Data 8).

## Discussion

This is the first study to demonstrate that ALS patients could have an anomalous decision‐making pattern, in which there is little exploration and infrequent shifts in their choice to other PRL task alternatives. ALS patients barely chose the option they deemed disadvantageous, but often changed their choice when they thought the value of the two alternatives was equal. P_αβ_ calculated by the Q‐learning model and logistic regression analysis well represented the unique choice pattern of patients with ALS. P_αβ_ was not correlated with age, education, other conventional cognitive tests, and disease severity. Network analysis using the ICC method demonstrated a significant correlation between P_αβ_ and decreased degree centrality in the regions associated with decision‐making (including the anterior cingulate gyrus and frontal pole). Altered decision‐making in ALS patients may be related to the decreased hub function of medial prefrontal areas.

### RL, Q‐learning model, inverse temperature, and learning rate

The PRL task was designed to assess RL capacity, but as Worthy et al^30^ demonstrated, participants often use heuristic‐based models, including the WSLS model, during these kinds of tasks. In particular, when working memory load was high, participants were more likely to adopt WSLS strategies than RL.^31^ In the WSLS model, they were more likely to stay‐with or shift‐to options with higher expected values than options with lower expected values (i.e., RL). The combined WSLS‐RL dual‐process model may provide a superior fit to the data relative to the WSLS model alone.^32^ When different learning parameters could be associated with positive and negative prediction errors^33^, ^34^ and participants had a high learning rate and high inverse temperature, decision‐making based on Q‐learning was close to that based on the WSLS model.

However, individual choices in the Q‐learning model are not made in isolation, but are embedded in a series of past experiences, decisions, and outcomes. Conversely, the WSLS model is only sensitive to the outcome of the previous choice. The mathematical formulas used for analysis are also different.^13^, ^25^, ^26^ We also thought the WSLS model was a different strategy to RL. Random choice behavior must also be treated as a different strategy from the RL strategy because random choice behavior indicates the failure of learning. Since the WSLS model and random choice are different strategies to the RL model, and the Q‐learning model should be applied to the participants who adopted RL, we used AIC to clarify which strategy (i.e., the RL, WSLS, and random choice models) was the most appropriate model and excluded participants classified as WSLS and random choice model. There were no significant differences between the proportion of WSLS to random choice between patients with ALS and control participants. We found that the unique choice behavior of ALS patients could be classified using the Q‐learning model. These findings supported the idea that the choice model is determined independently of disease state.

The Q‐learning model is the most commonly used RL algorithm for model‐free analysis of choice behavior. In the PRL task, the Q‐learning model provides two crucial parameters, that is, inverse temperature (beta) and learning rate (alpha). The beta value determines the randomness of choice behaviors. Patients with ALS had high beta values, indicating that they hardly shifted their choice to another alternative, especially in a good situation.^12^, ^13^ A significantly higher beta value could indicate that they were likely to select the higher value option, which might reflect stereotypic and compulsive behavior.

The alpha value has a complex relationship with performance in RL tasks; different learning environments afford distinct optimal learning rates. However, a low alpha value indicates difficulty in updating old values despite newly acquired information, and subsequently no shift in choice. In ALS patients with the unique choice pattern, the alpha value was significantly correlated with the total score and score at the reversal phase (Supplementary Data 6). This could be attributed to optimization of their internal model, indicating more flexible, goal‐directed actions due to representation of the contingencies of the task and less accelerated exploration, in addition to lower attention to the alternative choice after the situation changed.^35^, ^36^

### P_αβ_ indicates anomalous decision‐making behavior in patients with ALS

The balance between “exploration” for searching for a better choice and “exploitation” for obtaining a large reward is said to be one of the major issues in RL. ALS patients did not change their choice as frequently as controls (Figure 1), which means the change of the balance of exploration vs. exploitation trade‐off. ALS patients barely chose the option that they thought was disadvantageous, although they often changed their choice when they thought the value of the two alternatives had become equal. Their choice behavior seemed to be strictly guided by action values that are estimated through RL (such as Q‐learning). This anomalous choice behavior is the key point in the change in choice behavior in patients with ALS.

In the current study, we used P_αβ_, which was a better measure of anomalous PRL behavior in patients with ALS. The use of only alpha or beta values could not completely explain the uniqueness of choice behavior in patients with ALS. AIC showed P_αβ_ had stronger explanatory power than alpha or beta values alone. Interestingly, ICC analysis showed that P_αβ_ was significantly correlated with decreased degree centrality in the anterior cingulate gyrus and frontal pole in patients with ALS. This region was reportedly associated with the decision‐making process based on perceptual cues and reward values.^37^ Furthermore, patients with ALS frequently show pathological changes in these regions.^3^, ^38^ These findings support the idea that P_αβ_, induced by both alpha and beta values, can represent the anomalous decision‐making behavior related to the involvement of the prefrontal cortex in patients with ALS.

### Network alterations and altered decision‐making in patients with ALS

Seed‐based analysis from the anterior cingulate gyrus and frontal pole revealed changes in functional connectivity to regions that consist of the paracingulate gyrus, frontal medial cortex, anterior cingulate gyrus, frontal pole, subcallosal cortex, and superior frontal gyrus. The medial prefrontal cortex monitors whether choice behavior is reliable and enforces the switch from exploitation to exploration.^37^, ^39^ The functional connectivity of the frontal polar cortex alters when someone decides to switch to the alternative behavior, since the frontal pole plays a role in monitoring the alternative course of action.^37^, ^40^ The reorganization of these networks might lead to a change in the balance between exploitation and exploration, or influence the switch to the alternative behavior, subsequently producing the anomalous choice behavior in patients with ALS.

### Influence of motor dysfunction in patients with ALS on the PRL task

Upper and lower motor neurons are affected in patients with ALS, which could influence the selections made by pressing a key within a short period of time. Motor deficits might make ALS patients have a stronger choice perseverance tendency than control participants. However, we carefully assessed whether ALS patients performed the PRL task appropriately. We postulated that motor deficits could not significantly influence the uniqueness of choice behavior in ALS patients. There were no significant differences in the scores and strategies of the PRL task between ALS and control participants. Furthermore, the anomalous choice behavior linked with a high P_αβ_ required the selection of the advantageous stimulus, which was randomly presented on either the left or right side in each trial. Moreover, there was no significant correlation between P_αβ_ and disease duration, type, and severity (ALSFRS‐R). Thus, changes in choice behavior were not mainly due to motor dysfunction.

## Limitations

We classified the participants’ strategies into Q‐learning, WSLS, or random choice using only standard models. In standard RL models, the action values are assumed to be updated according to the reward prediction error. Numerous studies have noted that the magnitude of the update is biased depending on the sign of the reward prediction error.^33^, ^34^ The bias is represented in RL models by differential learning rates for positive and negative reward prediction errors.

Regarding the sensitivity of the choice probabilities shown by inverse temperature, choice perseverance, shown by choice trace weight “ϕ”,^25^, ^41^ more closely fitted ALS symptoms. Thus, in future work, it is recommended that other mathematical schemes be compared to reveal the anomalous choice behavior of ALS patients.

We found there were significant differences in cognitive function, especially memory, fluency, and language, between ALS patients and cognitively normal controls (Supplementary Data 2 and Table 1). Previous studies also showed ALS patients had frontotemporal dysfunction.^3^, ^42^ Our results supported the findings of these studies. However, the participants performed a practice task before the PRL task to ensure they knew how to perform the task. Besides, we could not find a significant correlation between P_αβ_ and conventional cognitive examinations. We postulated that cognitive dysfunction assessed by conventional examinations could not explain the anomalous choice behavior of patients with ALS.

Another limitation was that we assessed ICC with a cluster‐forming height threshold of *P* < 0.005. However, the contributing neural systems in our analysis overlapped with those found in task‐fMRI studies. Thus, it is best to perform RS‐fMRI analysis with more participants in a future study to obtain more reliable results.

## Conclusion

ALS patients showed anomalous changes in choice behavior during the PRL task. The Q‐learning model and logistic regression analysis were useful for quantifying the decision‐making process. Analysis of RS‐fMRI by ICC suggested that the anomalous changes in choice behavior in patients with ALS were associated with decreased degree centrality of medial prefrontal areas. Altered decision‐making in ALS patients may be related to the decreased hub function of these areas.

## Conflict of Interests

The authors declare no competing financial interests.

## Supporting information


**Supplementary Data 1.** Schematic representation of the probabilistic reversal learning task. A) Time course of a trial: The task comprises 120 trials. Following presentation of a cross‐hairs as a fixation on each trial, participants are presented two abstract line drawing on the left and right side of the fixation. Abstract stimuli were utilized to prevent participants from verbal coding and developing simple memory strategies. Subsequently participants choose one of the two stimuli by pressing a key within 1000 ms. After that, a feedback signal indicating either a reward (in Japanese “Atari”) or a loss (in Japanese “Hazure”) is presented. If the participant did not select a stimulus within the presentation time window, the message “Time‐up” (in Japanese “Jikangire”) appeared and the experiment continued. Stimulus material was run by Presentation (Neurobehavioral Systems, Albany, CA). B) Reward/loss ratio: During the first 60 trials, one stimuli is an advantageous option, in which reward/loss frequency ration was 80:20, whereas the other stimuli is a disadvantageous option, in which the reward/loss frequency is 20:80. The contingencies are reversed in the last 60 trials without any instruction to participants.

**Supplementary Data 2.** Clinical features and cognitive examinations and PRL task results in ALS patients and cognitively normal control participants. Data are shown as mean ± standard deviation (SD). Age, years of education, scores in cognitive examinations, and PRL task were compared by Mann–Whitney analysis. Sex was compared by chi‐squared test. The statistical significance threshold was set at *P* < 0.05. ACE‐R: Addenbrooke’s Cognitive Examination revised; MMSE: Mini‐Mental State Examination; PRL: Probabilistic Reversal Learning.
**Supplementary Data 4.** Correlation coefficient between Pαβ and age, education, disease duration, and conventional cognitive examinations. Pearson's rank correlation coefficient was performed to reveal the correlation between Pαβ and the clinical backgrounds. Spearman's rank correlation was performed to reveal the correlation between Pαβ and disease type. *: *P*‐value < 0.05, **: *P*‐value < 0.001.
**Supplementary Data 5.** Clinical backgrounds of patients with amyotrophic lateral sclerosis who had anomalous choice behavior and those who had typical choice behavior. Data are shown as mean ± standard deviation (SD). Age, education, disease duration, ALSFRS‐R, MMSE, ACE‐R, and PRL task were compared by Mann–Whitney analysis. Sex and clinical phenotype were compared by chi‐squared test. The statistical significance threshold was set at *P* < 0.05. ACE‐R: Addenbrooke’s Cognitive Examination revised; MMSE: Mini‐Mental State Examination; PRL: Probabilistic Reversal Learning.
**Supplementary Data 6.** Clinical features and cognitive examination and probabilistic reversal learning (PRL) task data of patients with amyotrophic lateral sclerosis (ALS) with a higher Pαβ. Pearson's correlation coefficient was performed to reveal the correlation between scores and the clinical backgrounds, parameters, P‐index, and conventional cognitive examinations. Only Sex and Disease Types were analyzed by Spearman's correlation. *: *P*‐value < 0.05, **: *P* ‐value < 0.001. MMSE: Mini‐Mental State Examination, ACE‐R: Addenbrooke’s Cognitive Examination revised.
**Supplementary Data 7.** Clinical backgrounds of patients with amyotrophic lateral sclerosis (ALS) who underwent magnetic resonance imaging (MRI) and did not undergo MRI. Data are shown as mean ± standard deviation (SD). Age, education, disease duration, ALSFRS‐R, MMSE, ACE‐R, and PRL task were compared by Mann–Whitney analysis. Sex and clinical phenotype were compared by chi‐squared test. The statistical significance threshold was set at *P* < 0.05. ACE‐R: Addenbrooke’s Cognitive Examination revised; MMSE: Mini‐Mental State Examination; PRL: Probabilistic Reversal Learning.

**Supplementary Data 3.** Selection of participants in the probabilistic reversal learning (PRL) Task.

**Supplementary Data 8.** Seed‐based analysis (SBA) in amyotrophic lateral sclerosis (ALS). SBA from the region of the anterior cingulate gyrus and frontal pole revealed that patients with ALS had decreased functional connectivity with the paracingulate gyrus, frontal medial cortex, anterior cingulate gyrus, frontal pole, subcallosal cortex, superior frontal gyrus.. The threshold was set at *P* < 0.005 for a cluster‐forming height threshold and an FDR‐corrected cluster‐size threshold of *P* <  0.05.
