# Supplementary figures and images for: The neural network basis of altered decision‐making in patients with amyotrophic lateral sclerosis
Source: Ann Clin Transl Neurol. 2020 Oct 22;7(11):2115–26. doi: 10.1002/acn3.51185 (PMC7664284; doi:10.1002/acn3.51185)

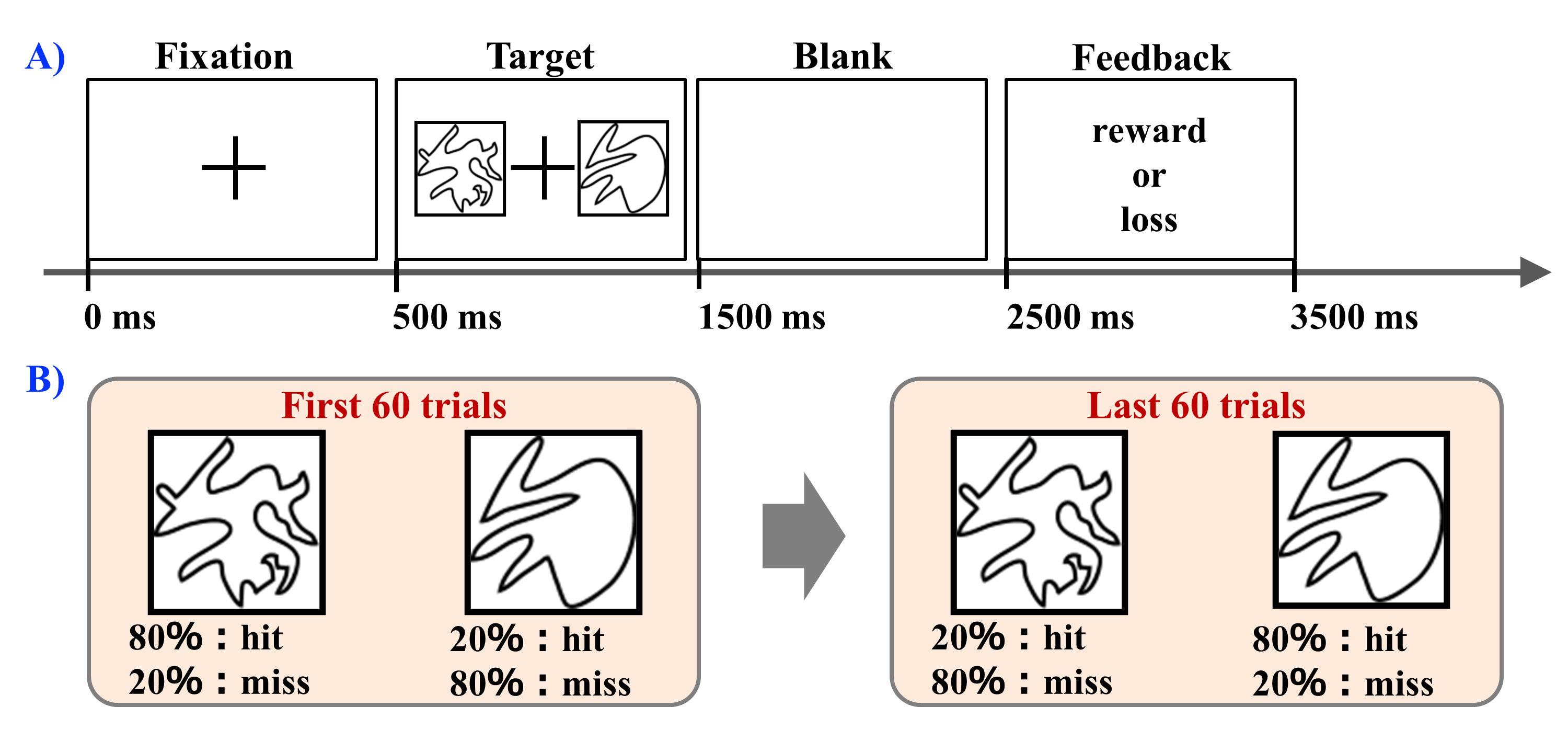

Supplement: Supplementary file 1 — Supplementary Data 1. Schematic representation of the probabilistic reversal learning task. A) Time course of a trial: The task comprises 120 trials. Following presentation of a cross‐hairs as a fixation on each trial, participants are presented two abstract line drawing on the left and right side of the fixation. Abstract stimuli were utilized to prevent participants from verbal coding and developing simple memory strategies. Subsequently participants choose one of the two stimuli by pressing a key within 1000 ms. After that, a feedback signal indicating either a reward (in Japanese “Atari”) or a loss (in Japanese “Hazure”) is presented. If the participant did not select a stimulus within the presentation time window, the message “Time‐up” (in Japanese “Jikangire”) appeared and the experiment continued. Stimulus material was run by Presentation (Neurobehavioral Systems, Albany, CA). B) Reward/loss ratio: During the first 60 trials, one stimuli is an advantageous option, in which reward/loss frequency ration was 80:20, whereas the other stimuli is a disadvantageous option, in which the reward/loss frequency is 20:80. The contingencies are reversed in the last 60 trials without any instruction to participants. [file ACN3-7-2115-s001.TIF]

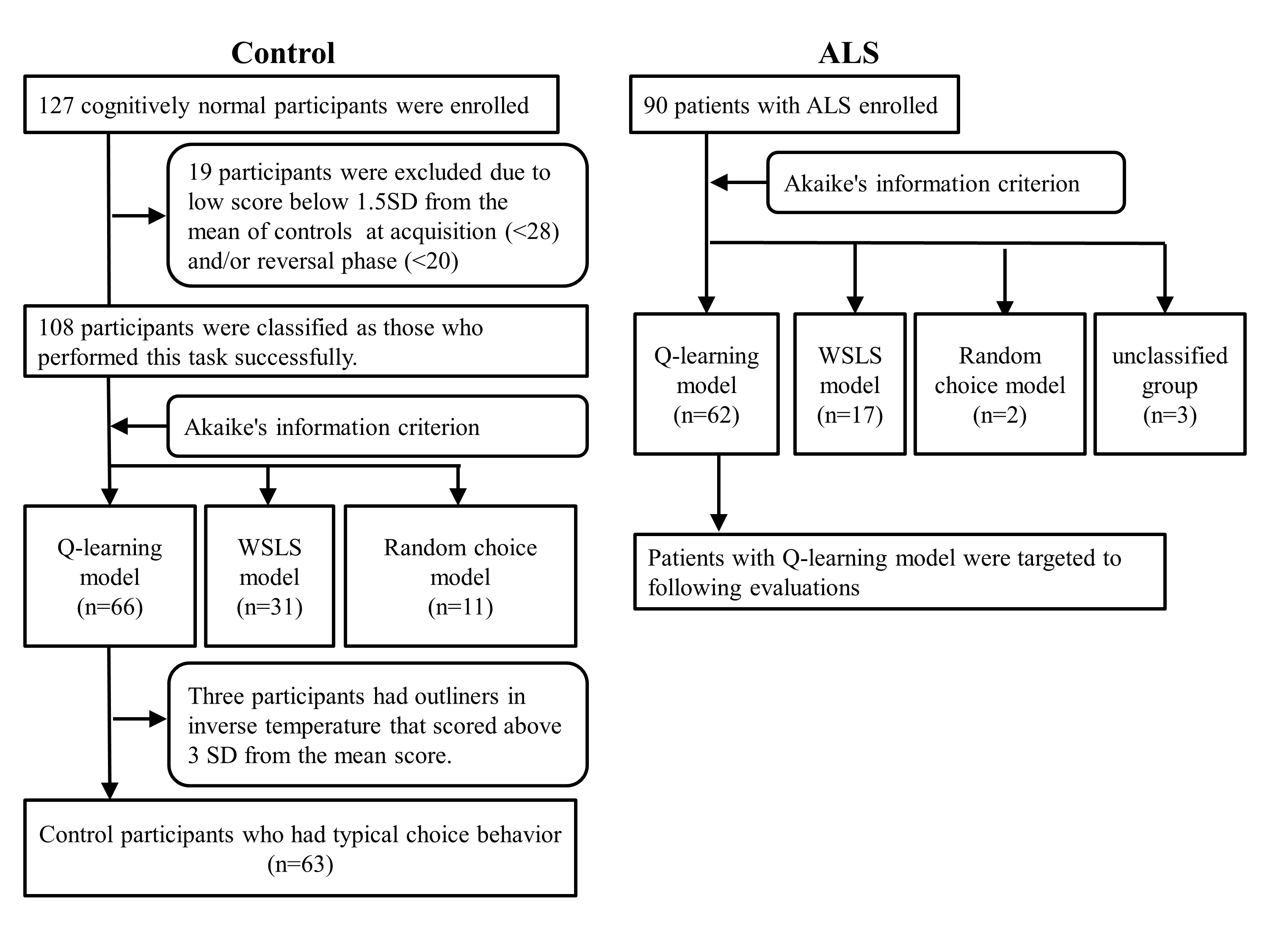

Supplement: Supplementary file 3 — Supplementary Data 3. Selection of participants in the probabilistic reversal learning (PRL) Task. [file ACN3-7-2115-s003.TIF]

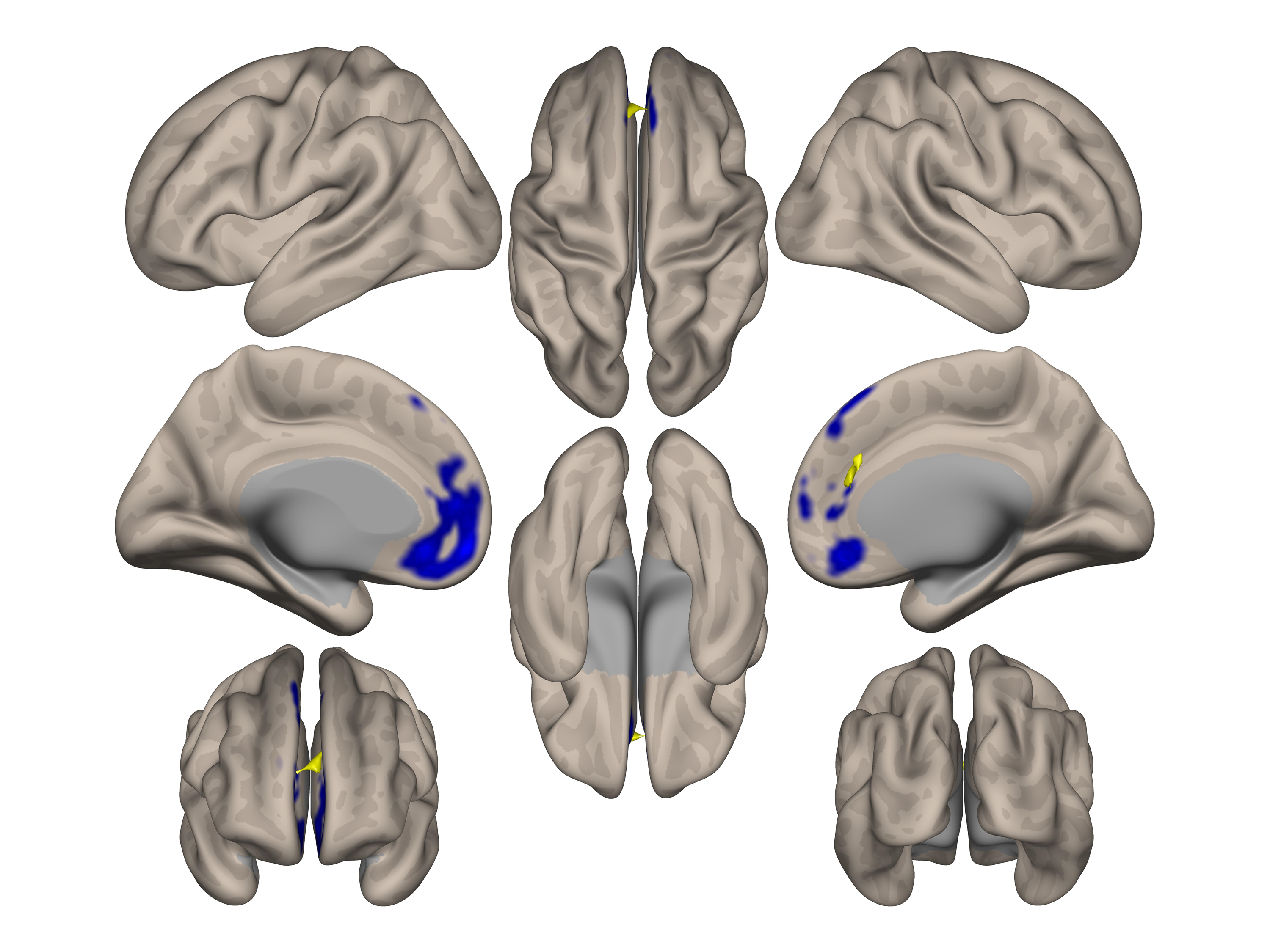

Supplement: Supplementary file 4 — Supplementary Data 8. Seed‐based analysis (SBA) in amyotrophic lateral sclerosis (ALS). SBA from the region of the anterior cingulate gyrus and frontal pole revealed that patients with ALS had decreased functional connectivity with the paracingulate gyrus, frontal medial cortex, anterior cingulate gyrus, frontal pole, subcallosal cortex, superior frontal gyrus.. The threshold was set at P < 0.005 for a cluster‐forming height threshold and an FDR‐corrected cluster‐size threshold of P < 0.05. [file ACN3-7-2115-s004.TIF]
