# Supplementary material for: The neural network basis of altered decision‐making in patients with amyotrophic lateral sclerosis
Source: Ann Clin Transl Neurol. 2020 Oct 22;7(11):2115–26. doi: 10.1002/acn3.51185 (PMC7664284; doi:10.1002/acn3.51185)
Supplement: Supplementary file 2 — Supplementary Data 2. Clinical features and cognitive examinations and PRL task results in ALS patients and cognitively normal control participants. Data are shown as mean ± standard deviation (SD). Age, years of education, scores in cognitive examinations, and PRL task were compared by Mann–Whitney analysis. Sex was compared by chi‐squared test. The statistical significance threshold was set at P < 0.05. ACE‐R: Addenbrooke’s Cognitive Examination revised; MMSE: Mini‐Mental State Examination; PRL: Probabilistic Reversal Learning. Supplementary Data 4. Correlation coefficient between Pαβ and age, education, disease duration, and conventional cognitive examinations. Pearson's rank correlation coefficient was performed to reveal the correlation between Pαβ and the clinical backgrounds. Spearman's rank correlation was performed to reveal the correlation between Pαβ and disease type. *: P‐value < 0.05, **: P‐value < 0.001. Supplementary Data 5. Clinical backgrounds of patients with amyotrophic lateral sclerosis who had anomalous choice behavior and those who had typical choice behavior. Data are shown as mean ± standard deviation (SD). Age, education, disease duration, ALSFRS‐R, MMSE, ACE‐R, and PRL task were compared by Mann–Whitney analysis. Sex and clinical phenotype were compared by chi‐squared test. The statistical significance threshold was set at P < 0.05. ACE‐R: Addenbrooke’s Cognitive Examination revised; MMSE: Mini‐Mental State Examination; PRL: Probabilistic Reversal Learning. Supplementary Data 6. Clinical features and cognitive examination and probabilistic reversal learning (PRL) task data of patients with amyotrophic lateral sclerosis (ALS) with a higher Pαβ. Pearson's correlation coefficient was performed to reveal the correlation between scores and the clinical backgrounds, parameters, P‐index, and conventional cognitive examinations. Only Sex and Disease Types were analyzed by Spearman's correlation. *: P‐value < 0.05, **: P ‐value < 0.001. MMSE: Mini‐M [file ACN3-7-2115-s002.docx]

Supplementary Data 2.

Clinical features and cognitive examinations and PRL task results in ALS patients and cognitively normal control participants.

Data are shown as mean ± standard deviation (SD). Age, years of education, scores in cognitive examinations and PRL task were compared by Mann-Whitney analysis. Sex was compared by chi-square test. The statistical significance threshold was set at p < 0.05. ACE-R: Addenbrooke’s Cognitive Examination revised; MMSE: Mini-Mental State Examination; PRL: Probabilistic Reversal Learning

|  | **Cognitively Normal Participants** | **ALS** | **P-value** |
| --- | --- | --- | --- |
| **No.** | **127** | **90** | **-** |
| **Age** | **64.5 (10.0)** | **65.0 (9.7)** | **N.S. (0.724)** |
| **Sex (men : women)** | **47:80** | **58:32** | **<0.001** |
| **Education (years)** | **13.7 (2.3)** | **12.1 (2.5)** | **<0.001** |
| **Clinical phenotype**  **(spinal: bulbar : others)** | **-** | **64:21:5** | **-** |
| **Disease duration (years)** | **-** | **1.9 (1.9)** | **-** |
| **ALSFRS-R** |  | **40.7 (4.7)** | **-** |
| **MMSE** | **29.4 (1.0)** | **27.7 (2.1)** | **<0.001** |
| **ACE-R Total score** | **97.1 (2.5)** | **88.3 (9.0)** | **<0.001** |
| **ACE-R Orientation/attention** | **17.9 (0.3)** | **17.5 (1.2)** | **<0.001** |
| **ACE-R Memory** | **24.7 (1.5)** | **20.3(4.6)** | **<0.001** |
| **ACE-R Fluency** | **13.6 (1.0)** | **11.9 (2.7)** | **<0.001** |
| **ACE-R Language** | **25.3 (1.0)** | **23.7 (2.3)** | **<0.001** |
| **ACE-R visuospatial abilities** | **15.7 (0.7)** | **15.0 (1.2)** | **<0.001** |
| **Total score in PRL task** | **68.8 (10.1)** | **67.7 (11.7)** | **N.S. (0.576)** |
| **Score at acquisition phase** | **38.1 (6.9)** | **39.1 (7.7)** | **N.S. (0.117)** |
| **Score at reversal phase** | **30.8 (6.9)** | **28.6 (9.1)** | **N.S. (0.094)** |
| **Q-learning vs WSLS vs R** | **74:36:16** | **62:20:5** | **N.S. (0.111)** |

Supplementary Data 4.

Correlation coefficient between Pαβ and age, education, disease duration, and conventional cognitive examinations

| **Pearson** | **Total score**  **in PRL task** | **Score**  **at acquisition phase** | **Score**  **at reversal phase** | **Alpha** | **Beta** | **P**_αβ_ |
| --- | --- | --- | --- | --- | --- | --- |
| **Age** | -0.125 | -0.160 | -0.017 | 0.063 | -0.038 | -0.119 |
| **Education (years)** | -0.095 | -0.110 | -0.022 | 0.008 | 0.020 | -0.012 |
| **Disease duration (years)** | 0.013 | 0.070 | -0.030 | -0.044 | -0.099 | -0.146 |
| **ALSFRSR** | 0.189 | 0.102 | 0.131 | .287^*^ | 0.096 | -0.029 |
| **MMSE** | .364^**^ | 0.171 | .280^*^ | 0.226 | -0.070 | -0.162 |
| **ACE-R Total Score** | 0.245 | 0.106 | 0.196 | 0.257 | 0.025 | -0.112 |
| **ACE-R Orientation/Attention** | .328^*^ | 0.189 | 0.226 | 0.154 | 0.042 | -0.020 |
| **ACE-R Memory** | 0.253 | 0.066 | 0.236 | .268^*^ | -0.062 | -0.197 |
| **ACE-R Fluency** | 0.069 | 0.118 | -0.014 | 0.005 | 0.124 | 0.104 |
| **ACE-R Language** | 0.062 | -0.047 | 0.107 | .259^*^ | 0.045 | -0.110 |
| **ACE-R Visuospatial Abilities** | .324^*^ | 0.200 | 0.212 | .329^*^ | 0.030 | -0.123 |
| **FAB** | .321^*^ | 0.043 | .336^**^ | 0.166 | -0.014 | -0.146 |
| **Verbal Fluency (letter)** | 0.030 | 0.001 | 0.034 | -0.129 | 0.059 | 0.086 |
| **Verbal Fluency (category)** | 0.101 | -0.041 | 0.147 | -0.045 | -0.011 | -0.028 |
| **Digit Span Forward** | -0.235 | -.506^**^ | 0.128 | -0.017 | 0.162 | 0.088 |
| **Digit Span Backward** | -0.095 | -0.110 | -0.022 | 0.008 | 0.020 | -0.012 |
| **Stroop Test** | 0.013 | 0.070 | -0.030 | -0.044 | -0.099 | -0.146 |

| **Spearman** | **Total score**  **in PRL task** | **Score**  **at acquisition phase** | **Score**  **at reversal phase** | **Alpha** | **Beta** | **P**_αβ_ |
| --- | --- | --- | --- | --- | --- | --- |
| **Disease Type** | -0.009 | 0.061 | -0.094 | 0.151 | -0.105 | -0.216 |

Pearson's rank correlation coefficient was performed to reveal the correlation between Pαβ and the clinical backgrounds.

Spearman's rank correlation was performed to reveal the correlation between Pαβ and disease type.

*: p-value < 0.05, **: p-value < 0.001

Supplementary Data 5.

Clinical backgrounds of patients with amyotrophic lateral sclerosis who had anomalous choice behavior and those who had typical choice behavior.

|  | **ALS patients with typical choice behavior** | **ALS patients with anomalous choice behavior** | **P_αβ_** |
| --- | --- | --- | --- |
| **N** | **27** | **35** | **-** |
| **Age** | **66.6 (7.4)** | **64.7 (9.8)** | **N.S. (0.162)** |
| **Sex** | **18:9** | **23:12** | **N.S. (0.937)** |
| **Education (years)** | **12.2 (2.8)** | **12.0 (2.5)** | **N.S. (0.929)** |
| **Clinical phenotype (Spinal : Bulbar : others)** | **18:8:1** | **26:8:1** | **N.S. (0.807)** |
| **Disease duration (years)** | **2.0 (1.2)** | **1.7 (1.5)** | **N.S. (0.101)** |
| **ALSFRS-R** | **41.1 (3.9)** | **40.7 (4.4)** | **N.S. (0.765)** |
| **MMSE** | **28.0 (1.8)** | **27.5 (2.4)** | **N.S. (0.472)** |
| **ACE-R** | **90.1 (9.2)** | **88.2 (8.5)** | **N.S. (0.274)** |
| **Total Score in PRL task** | **72.2 (7.5)** | **72.5 (10.8)** | **N.S. (0.865)** |
| **Score at acquisition phase** | **38.5 (4.9)** | **42.7 (7.0)** | **<0.001** |
| **Score at reversal phase** | **33.7 (5.4)** | **29.7 (9.5)** | **N.S. (0.165)** |
| **Alpha** | **0.4 (0.3)** | **0.26 (0.3)** | **0.022** |
| **Beta** | **3.1 (0.9)** | **9.9 (8.4)** | **< 0.001** |
| **P_αβ_** | **0.39 (0.1)** | **0.68 (0.2)** | **< 0.001** |

Data are shown as mean ± standard deviation (SD). Age, education, disease duration, ALSFRS-R, MMSE, ACE-R and PRL task were compared by Mann-Whitney analysis. Sex and clinical phenotype were compared by chi-square test. The statistical significance threshold was set at p < 0.05. ACE-R: Addenbrooke’s Cognitive Examination revised; MMSE: Mini-Mental State Examination; PRL: Probabilistic Reversal Learning

Supplementary Data 6

Clinical features and cognitive examination and probabilistic reversal learning (PRL) task data of patients with amyotrophic lateral sclerosis (ALS) with a higher Pαβ

| **Pearson** | **Age** | **Sex** | **Education** | **Disease**  **Type** | **Duration** | **ALSFRS-R** | **Alpha** | **Beta** | **P_αβ_** | **MMSE** | **ACE-R** |
| --- | --- | --- | --- | --- | --- | --- | --- | --- | --- | --- | --- |
| **Total**  **Score** | **-0.131** | **0.069** | **-0.129** | **0.134** | **-0.043** | **0.136** | **0.695^**^** | **0.124** | **-0.067** | **0.568^**^** | **0.404^*^** |
| **Score at acquisition phase** | **-0.139** | **0.120** | **-0.099** | **0.197** | **0.02** | **0.204** | **0.248** | **-0.243** | **-0.153** | **0.347^*^** | **0.334** |
| **Score at reversal phase** | **-0.046** | **-0.048** | **-0.073** | **-0.028** | **-0.057** | **-0.009** | **0.604^**^** | **0.319** | **0.037** | **0.380^*^** | **0.206** |

Pearson's correlation coefficient was performed to reveal the correlation between scores and the clinical backgrounds, parameters, P-index, and conventional cognitive examinations.

Only Sex and Disease Types were analyzed by Spearman's correlation.

*: p-value < 0.05, **: p-value < 0.001.

MMSE:Mini-Mental State Examination, ACE-R:Addenbrooke’s Cognitive Examination revised

Supplementary Data 7

Clinical backgrounds of patients with amyotrophic lateral sclerosis (ALS) who underwent magnetic resonance imaging (MRI) and did not undergo MRI.

|  | **ALS with MRI n=34** | **ALS without MRI n=28** | **P-value** |
| --- | --- | --- | --- |
| **Age** | **67.7 (7.7)** | **63.8 (10.2)** | **N.S.** |
| **Sex** | **23:11** | **18:10** | **N.S.** |
| **Education (years)** | **11.9 (2.8)** | **12.3 (2.2)** | **N.S.** |
| **Clinical phenotype (Spinal : Bulbar : others)** | **24:9:1** | **20:7:1** | **N.S.** |
| **Disease duration (years)** | **1.9 (1.3)** | **1.8 (1.4)** | **N.S.** |
| **ALSFRS-R** | **41.4 (3.6)** | **40.2 (4.4)** | **N.S.** |
| **MMSE** | **27.8 (1.7)** | **27.5 (2.6)** | **N.S.** |
| **ACE-R** | **91.3 (7.4)** | **86.3 (9.7)** | **0.039** |
| **Total Score in PRL task** | **73.3 (8.4)** | **71.2 (10.6)** | **N.S.** |
| **Score at**  **acquisition phase** | **41.4 (5.0)** | **40.3 (8.0)** | **N.S.** |
| **Score at**  **reversal phase** | **31.9 (7.6)** | **30.9 (9.0)** | **N.S.** |
| **Alpha** | **0.40 (0.3)** | **0.25 (0.3)** | **N.S.** |
| **Beta** | **6.5 (6.4)** | **7.6 (8.1)** | **N.S.** |
| **P_αβ_** | **0.53 (0.2)** | **0.58 (0.2)** | **N.S.** |

Data are shown as mean ± standard deviation (SD). Age, education, disease duration, ALSFRS-R, MMSE, ACE-R and PRL task were compared by Mann-Whitney analysis. Sex and clinical phenotype were compared by chi-square test. The statistical significance threshold was set at p < 0.05. ACE-R: Addenbrooke’s Cognitive Examination revised; MMSE: Mini-Mental State Examination; PRL: Probabilistic Reversal Learning
